# Skodaïc Resonance in Lobar Pneumonia

**Published:** 1909-04-03

**Authors:** 


### Medicine.

### SKODAÏC RESONANCE IN LOBAR PNEUMONIA.

Since Skoda first drew attention to the fact that the percussion note over the upper part of the front of the chest may be tympanitic or higher pitched than that of the other or healthy side when there is a pleuritic effusion or empyema at the base, so-called "Skodaïc resonance" has been well enough known as one of the physical signs of pleural effusion. It is, perhaps, less generally known that Skodaïc resonance precisely similar to that found in connection with effusions may also occur in certain cases of lobar pneumonia where there is no effusion at all. The occurrence of Skodaïc resonance over the upper lobe in front, in a case of lobar pneumonia, is no proof of there being effusion in addition to pneumonia. The following is an illustrative case:—

A young soldier, aged 22, was admitted to hospital, on December 12, for an illness that had begun three days previously with a rigor and acute pain in the left side of his chest. His other symptoms were dyspnœa, short irritating cough, thirst, loss of appetite, and a feeling of being so ill that he must stay in bed. There is little need to give the details of his illness beyond saying that he had the typical pyrexia, pulse and respiration rates of lobar pneumonia, and also typical rusty sputum. It is with the physical signs that we propose to deal here. On December 12 there was nothing abnormal to be made out in the right lung; over the left, at the very base posteriorly, there were impairment of percussion note and deficiency of breath sounds, with faint bronchial breathing and bronchophony, but no crepitations. Tactile vocal fremitus seemed, if anything, to be diminished at the left base. As regards the upper part of the left lung, both behind and in front, the physical signs seemed normal. On December 13 these physical signs were precisely similar, except that fine crepitations were occasionally to be made out in the area at the left base where there was bronchial breathing. The diagnosis of lobar pneumonia was made definitely. On December 14 and 15 the signs were similar but more extensive. Bronchial breathing could be heard over the whole of the left lung posteriorly, whilst in front the left lung was resonant, and the compensatory type of vesicular murmur was to be heard in it. On December 16 the patient was considerably more ill, expectoration was abundant and characteristic. All over the back of the left lung there was bronchial breathing, with here and there a patch of crepitant râles: it was quite dull to percussion from apex to base. Similar signs extended into the left axilla, but in front there was increased resonance from clavicle to nipple, with less marked bronchial breathing here than behind. The right lung still gave perfectly natural physical signs. By December 18 the resonance over the upper part of the left lung in front had increased to such a degree that it was absolutely tympanitic below the clavicle. The percussion sound was as well marked a Skodaïc note as one ever hears in a case of pleuritic effusion. There was bronchial breathing to be heard over every part of the left lung except over the region of Skodaïc resonance, and it was thought that the upper part of the left upper lobe still contained vesicular air, whilst all the rest was in a state of hepatisation. The Skodaïc note, as well marked as ever, persisted until the patient's death on December 21. The only change that occurred in the physical signs between December 18 and 21 was the development of crepitations and bronchial breathing over the back of the right lung on December 20.

At the post-mortem examination the heart, pericardium, stomach, and abdominal organs were all healthy; the only obvious lesions were in the lungs and pleuræ. There was no pleuritic effusion, but the whole of the left lung was in a state of grey hepatisation from apex to base, without a single portion being left capable of floating in water; the right lower lobe had become recently hepatised posteriorly, and there was acute pleurisy with yellow exudate over the hepatised regions.

The patient, therefore, came to hospital for a lobar pneumonia of the left side, which extended from apex to base. During the last few days of his life there was a very marked tympanitic or Skodaïc note over the upper part of the left upper lobe in front, which seemed to indicate that the left upper lobe was not completely consolidated. The patient died whilst the physical signs were still the same, and the autopsy showed that the left lung was hepatised throughout; and that this condition had existed for several days was indicated by the fact that the colour of the hepatisation was grey.

Those who have written about Skodaïc resonance in lobar pneumonia without effusion have usually assumed that the physical sign is due either to a partial pneumothorax, or to the upper lobe not being consolidated at the same time as the lower. Such explanations will not account for the case described, however. It is clear that Skodaïc resonance can occur in lobar pneumonia without effusion, even when the entire lung has been rendered airless by hepatisation. This is an important point to realise in the interpretation of the physical signs in any given case. The most likely explanation of the tympanitic note in the subclavicular region would seem to be that percussion evokes the resonance of the big bronchi and the termination of the trachea, so that it corresponds with the tracheal sound which

Dr. Williams drew attention to in normal children as being sometimes present on either side of the sternum, particularly the left.

This raises the question of what is the state of the lung in cases of pleuritic effusion in which Skodaïc resonance near the apex is well marked. It would seem that, rather than meaning that the upper lobe still contains air, it may signify a considerably greater degree of compression by the effusion. Dr. Laveran more than once observed that in cases of pleuritic effusion, where during life the phenomenon of Skodaïc resonance was particularly well marked, post-mortem examination showed that the lung on the affected side was in contact with the anterior chest wall, but absolutely airless throughout owing to compression. It seems, therefore, that in some of these cases also the tympanitic or Skodaïc note in the subclavicular region may be due, not to air in the upper lobe, but to indirect percussion of the trachea and large bronchi, the consolidated pulmonary parenchyma then playing the part of a pleximeter between the big bronchi and the chest wall.

